# Immunosenescence: molecular mechanisms and diseases

**DOI:** 10.1038/s41392-023-01451-2

**Published:** 2023-05-13

**Authors:** Zaoqu Liu, Qimeng Liang, Yuqing Ren, Chunguang Guo, Xiaoyong Ge, Libo Wang, Quan Cheng, Peng Luo, Yi Zhang, Xinwei Han

**Affiliations:** 1 Department of Interventional Radiology, The First Affiliated Hospital of Zhengzhou University, 450052 Zhengzhou, Henan China; 2 Interventional Institute of Zhengzhou University, 450052 Zhengzhou, Henan China; 3 Interventional Treatment and Clinical Research Center of Henan Province, 450052 Zhengzhou, Henan China; 4 Nephrology Hospital, the First Affiliated Hospital of Zhengzhou University, Zhengzhou University, 4500052 Henan, China; 5 Department of Respiratory and Critical Care Medicine, The First Affiliated Hospital of Zhengzhou University, 450052 Zhengzhou, Henan China; 6 Department of Endovascular Surgery, The First Affiliated Hospital of Zhengzhou University, 450052 Zhengzhou, Henan China; 7 Department of Hepatobiliary and Pancreatic Surgery, The First Affiliated Hospital of Zhengzhou University, 450052 Zhengzhou, Henan China; 8 Department of Neurosurgery, Xiangya Hospital, Central South University, Changsha, China; 9 Department of Oncology, Zhujiang Hospital, Southern Medical University, Guangzhou, China; 10 Biotherapy Center and Cancer Center, The First Affiliated Hospital of Zhengzhou University, 450052 Zhengzhou, China

**Keywords:** Cancer microenvironment, Senescence

## Abstract

Infection susceptibility, poor vaccination efficacy, age-related disease onset, and neoplasms are linked to innate and adaptive immune dysfunction that accompanies aging (known as immunosenescence). During aging, organisms tend to develop a characteristic inflammatory state that expresses high levels of pro-inflammatory markers, termed inflammaging. This chronic inflammation is a typical phenomenon linked to immunosenescence and it is considered the major risk factor for age-related diseases. Thymic involution, naïve/memory cell ratio imbalance, dysregulated metabolism, and epigenetic alterations are striking features of immunosenescence. Disturbed T-cell pools and chronic antigen stimulation mediate premature senescence of immune cells, and senescent immune cells develop a proinflammatory senescence-associated secretory phenotype that exacerbates inflammaging. Although the underlying molecular mechanisms remain to be addressed, it is well documented that senescent T cells and inflammaging might be major driving forces in immunosenescence. Potential counteractive measures will be discussed, including intervention of cellular senescence and metabolic-epigenetic axes to mitigate immunosenescence. In recent years, immunosenescence has attracted increasing attention for its role in tumor development. As a result of the limited participation of elderly patients, the impact of immunosenescence on cancer immunotherapy is unclear. Despite some surprising results from clinical trials and drugs, it is necessary to investigate the role of immunosenescence in cancer and other age-related diseases.

## Introduction

**Fig. 1:**
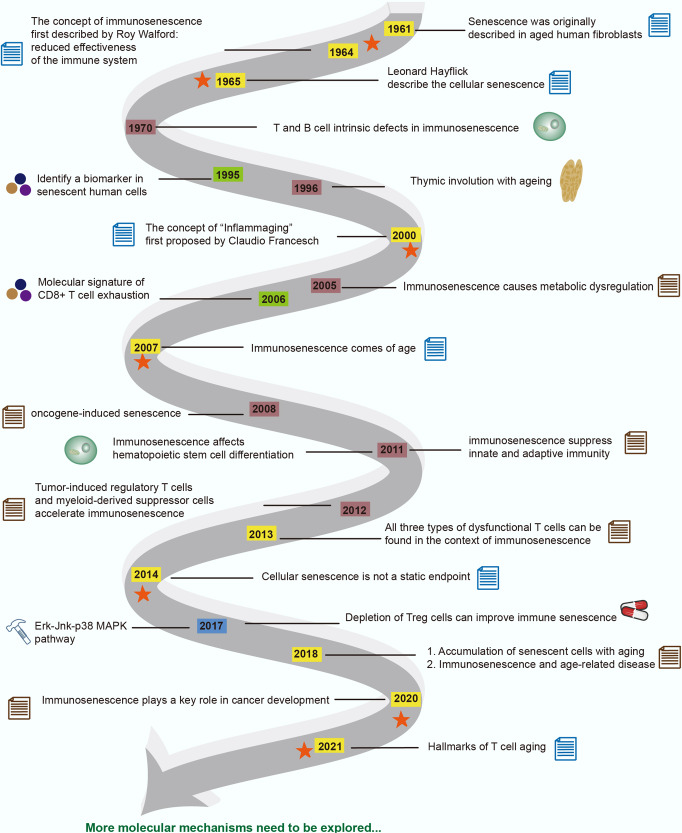
A timeline of events in the research history of immunosenescence. In this figure, we summarize the concept regarding immunosenescence, the breakthrough discovery of molecular mechanisms and biomarkers, and draw a timeline of research history

The increase in human life expectancy has led to a concomitant aging of the population, who are not only at higher risk for age-related diseases, including tumors, but also for a higher failure rate of immunotherapy and an increase in the recurrence rate after treatment.^1,2^ This phenomenon is known as immunosenescence and was first described by Roy Walford.^3–7^ Immunosenescence has defined the destruction and remodeling of immune organ structure as well as innate and adaptive immune dysfunction with aging, causing poor vaccination outcomes, and increased susceptibility to infection, age-related disease, and malignancies.^8–11^ Since the 1980s, scientists have begun to delve deeper into the mechanisms and effects of immunosenescence, and have found some important research results. Age-related declines in coping capacity and concomitant increases in proinflammatory status are the hallmarks of immunosenescence. This phenomenon, known as “inflammaging”, first proposed by Claudio Franceschi in 2000,^12^ is caused by constant antigen load and stress.^12^ In addition, thymic involution has been one of the most dramatic and ubiquitous changes.^13^ Immunosenescence affects both the innate and adaptive immune systems and certain immune cell types are more affected.^14^ In both mouse and human models, the hematopoietic stem cell (HSC) population of aged individuals had an increased frequency of immunophenotypes, was less quiescent, and showed myeloid-biased potentials, with transcriptional upregulation of genes related to cell cycle, myeloid-biased differentiation and myeloid malignancies.^15^ Attenuated acquired immunity is mainly associated with dwindling T-cell output, a remarkable characteristic of immunosenescence.^16^ The paradoxical coexistence of a massive influx of hyporesponsive tumor-infiltrating lymphocytes (TILs) in the tumor microenvironment (TME) and continued tumor aggressiveness also implies that dysfunctional T cells may hinder a successful antitumor response and immunotherapy.^17–19^ During immune system aging, three dysfunctional T-cell types can be observed: exhausted, senescent, and aged T cells.^20,21^ Among them, senescent T cells are the most studied. Senescence was originally described as a phenomenon in aged human fibroblasts both in culture and in vivo^22^; however, few markers were found in common between normal aging and senescence. Cellular senescence is associated with numerous cellular processes of aging, and the accumulation of senescent cells is one of the key pathological features associated with aging. Senescent cells are characterized by permanent cell cycle arrest accompanied by morphological abnormalities, loss of proliferative ability, and resistance to apoptosis.^23,24^ As a physiological response, it prevents genomic instability induced by persistent antigens and therefore the accumulation of DNA damage.^25^ However, excessive senescence, particularly of T cells, may ultimately lead to catastrophic immune decline and an increased risk of age-related diseases.^26,27^ Cellular senescence can be divided into premature senescence and telomere-dependent senescence (age-dependent replicative senescence).^23,24,28–30^ The senescence discussed in this review primarily refers to premature senescence triggered by damage signals such as oxidative stress, mitochondrial dysfunction, epigenetic changes, cytokines, perturbed proteostasis, persistent DNA damage, and mitogenic oncogenes. We summarize the significant findings in the research history of immunosenescence and illustrate the Fig. 1.

Given the dilemma of rising health care costs resulting from aging populations across the globe, gaining a comprehensive understanding of the intricate mechanisms underlying immunosenescence and senescent T cells could lead to the exploration of more effective therapeutic strategies. In this article, we focus on outlining the features of immunosenescence, its specific molecular mechanisms, and associated diseases, with particular attention to senescent T cells. We delve into the extensive metabolic and epigenetic pathways that determine T-cell fate and senescence. Additionally, we compared exhausted, senescent, and aged T cells as well as several clinical trials that have involved senescent immune cells. Based on these results, we propose possible interventional strategies targeting the metabolic-epigenetic axis of immunosenescence and senescent T cells to enhance current immunotherapy and extend life expectancy.

## Concept and hallmarks of immunosenescence

**Fig. 2:**
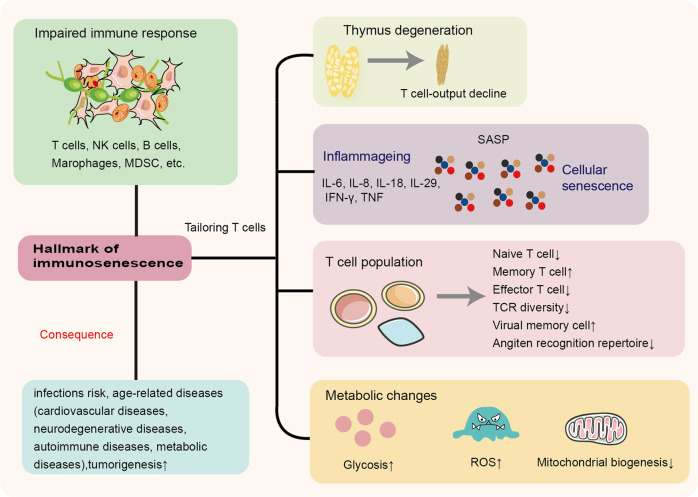
Hallmarks of immunosenescence and related diseases. Various immune cell subsets changed during immunosenescence. There were significant changes in T-cell subpopulations, including a decline in T-cell production associated with age due to thymic degeneration, abnormal T-cell metabolism, changes in the proportion of T subpopulations, and an SASP-mediated chronic low-grade inflammatory environment. IFN-γ interferon-γ, IL-6 interleukin-6, IL-8 interleukin-8, IL-18 interleukin-18, IL-29 interleukin-29, MDSC myeloid-derived suppressor cells, NK cells natural killer cells, ROS reactive oxygen species, SASP senescence-associated secretory phenotype, TCR T-cell receptor, TNF tumor necrosis factor

Immunosenescence is a complex process that involves organ reorganization and numerous regulatory processes at the cellular level.^31^ As a result, immune system function decreases, leading to an inadequate response to infections or vaccines in elderly individuals. Although the full extent of the biological changes is unknown, several characteristic changes are typically observed such as thymic involution, HSC dysfunctions, disrupted naïve/memory ratio in T and B cells, inflammaging, accumulation of senescent cells, impaired new antigen response, mitochondrial dysfunction, genomic instability, and stress responses^32–35^ (Fig. 2). Identifying hallmarks and characteristics associated with immunosenescence is crucial for exploring its impact and significance, particularly in age-related diseases.

Thymic involution plays a vital role in the imbalance of immune cell proportions, particularly for T cells.^36–38^ Thymic tissue can be divided into epithelial tissue and nonepithelial perivascular space without thymopoiesis. As the thymus atrophies, the epithelial spaces gradually disappear, and the perivascular space gradually fills the elderly thymus, leading to a decrease in naïve T cells, an increase in peripheral late-differentiated memory T cells, and diminished migration of naïve T cells to the periphery.^39–41^ Recent research has found that young adults who underwent thymectomy in early childhood for congenital heart disease displayed premature immunosenescence compared with age-matched adults, with altered T-cell profiles commonly seen in elderly individuals. This provides much-needed evidence for the role of thymic involution in human immune system aging.^42^ According to the latest studies, thymic rejuvenation does not restore diversity, and we agree that thymic degeneration does not perfectly explain the decline in T-cell receptor (TCR) diversity in humans.^43,44^

One of the hallmarks of immunosenescence is “inflammaging,” which refers to a systemic state of chronic low-grade inflammation characterized by upregulated blood inflammatory markers and is considered the central pillar of aging.^31,45^ The accumulation of damaged macromolecules is responsible for inflammaging, and endogenous host-derived cell debris is the source of chronic tissue damage.^46^ Cellular senescence is central to the inflammaging process, and Effros RB et al. found that senescent CD8^+^ cells accumulated in the context of immunosenescence in vivo.^47^ Senescent cells exhibit a distinctive senescence-associated secretory phenotype (SASP) that secretes a plethora of soluble factors, including interleukin-1 (IL-1), IL-6, IL-8, IL-13, IL-18, and tumor necrosis factor (TNF) and its receptors, leading to the inflammaging phenotype.^48–51^ Cellular senescence is a classic example of antagonistic pleiotropy, in which a specific gene trait elicits a phenotype that is beneficial early in life, for example, generating a well-recognized protective barrier to prevent tumorigenesis, which is essential for normal embryonic development and tissue repair.^52^ However, later in life, this phenotype becomes detrimental in an aged organism.^52–54^ Cellular senescence has been hotly debated as a driver of immunosenescence.

As the immune system ages, metabolism undergoes changes that involve increased glycolysis, mitochondrial dysfunction, and reactive oxygen species (ROS).^55,56^ These features of immunosenescence are strongly related to high morbidity and mortality from age-associated diseases such as cardiovascular diseases, neurodegenerative diseases, autoimmune diseases, metabolic diseases, and cancers in older patients.^57,58^ As the incidence of these disorders exponentially increases later in life, common cellular and molecular mechanisms likely contribute to their development.^53^ In this context, it is crucial to examine the molecular mechanisms, altered immune cell pool, and regulatory signaling impact on immunosenescence and age-related diseases.

## Aberrant molecular mechanisms within immunosenescence

In addition to aging, numerous factors such as chronic inflammation, cellular senescence, and the TME can also modulate the process of immunosenescence.^9,31^ Exposure to ultraviolet radiation, alcohol, smoking, pollution, and lack of exercise further contribute to immunosenescence.^1^ The entire pathway of immunosenescence remains to be fully elucidated. Considering that the complex physiological phenotypes exhibited during immunosenescence in vivo are the result of synergistic and antagonistic changes in multiple pathways, all possible therapies aimed at nonspecific “restoration” of the immune system might be counterproductive. Therefore, it is necessary to identify possible mechanistic targets and perform targeted interventions to safely “restore” the immune status of older adults.^59^ The mechanisms regulating immunosenescence are broadly attributed to damage, inflammaging, epigenetic remodeling, persistent antigen stimulation, thymic involution, and cellular senescence.

### Persistent antigen stimulation and stress-induced damage

**Fig. 3:**
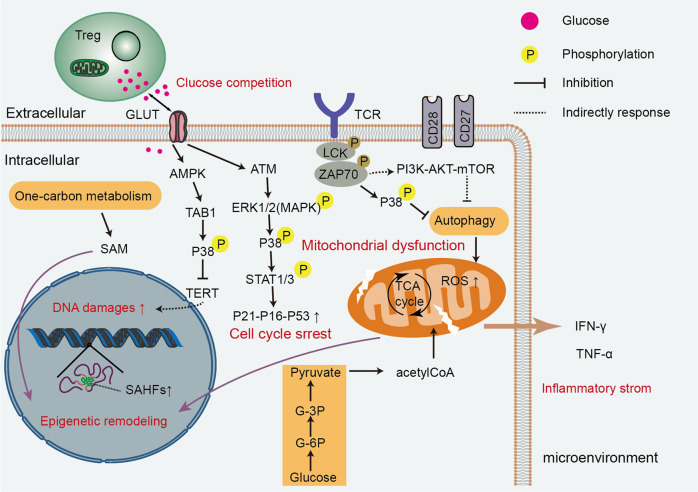
Multiple pathways are involved in T-cell senescence. Cellular senescence can be initiated by genomic or epigenomic damage that is induced by persistent antigens and competition for scarce nutrients in the tumor microenvironment. These stimuli lead to the activation of p38, ultimately triggering a DNA damage response, cell proliferation arrest, and inhibition of autophagy. Existing evidence suggests a potential metabolism-epigenetic axis that regulates T-cell senescence, in which mitochondrial function plays an important mediating role. AKT protein kinase B, AMPK adenosine 5′monophosphate-activated protein kinase, ERK extracellular-signal-regulated protein kinase, GLUT glucose transporters, IFN-γ interferon-γ, LCK lymphocyte-specific protein tyrosine kinase, MAPK mitogen-activated protein kinase, mTOR mechanism target of rapamycin, PI3K phosphatidylinositol 3-kinase, ROS reactive oxygen species, SAHFs senescence-associated heterochromatic focis, SAM S-adenosylmethionine, STAT1/3 signal transducer and activator of transcription 1/3, TAB1 transforming growth factor-activated kinase binding protein 1, TCA cycle tricarboxylic acid cycle, TERT telomerase reverse transcriptase, TNF-α tumor necrosis factor α, Treg regulatory T cells, ZAP70 zeta-chain-associated protein kinase 70

Numerous discoveries have generated a consensus that the antigen burden to which individuals are exposed during their lifetime is linked to immunosenescence.^60^ Khan et al. found that latent cytomegalovirus-infected individuals have a reduced pool of naïve and early memory T cells and clonal expansions of CD8^+^ T cells with a CD28^-^CD57^+^ phenotype, resulting in a decline in T-cell responses.^61,62^ Similar T-cell alterations have been identified in the TME of multiple tumor types. The proportion of tumor-associated regulatory T (Treg) cells is increased in the TME,^63^ and hypoxia-driven accumulation of cyclic adenosine monophosphate can inhibit tumor-specific effector T cells, activating p38 pathways.^64–67^ Mechanistically, competition for glucose between upregulated Tregs and effector T cells triggers ATM-related DNA damage, induces extracellular regulated protein kinases 1/2 (ERK1/2) and p38 pathways, and activates signal transducer and activator of transcription 1/3 (STAT1/3) with upregulation of p21-p16-p53, eventually causing T cells to stably withdraw from the cell cycle.^63,65^ Adenosine 5′monophosphate-activated protein kinase (AMPK) pathways activated by glucose deprivation could downregulate telomerase reverse transcriptase gene binding to transforming growth factor-activated protein kinase binding protein 1 (TAB1), causing autophosphorylation of p38, which in turn initiates T-cell senescence and DNA damage (Fig. 3).^68^ Moreover, lifelong chronic antigen loading also leads to T lymphocyte population exhaustion to fill the immune space, exacerbating the reduced T-cell repertoire.^69^ This suggests that distorted T-cell pools resulting from immunosenescence can be accelerated by long-term latent antigens in elderly individuals.^70^ Chronic antigenic stress triggers an inflammatory status via progressive activation of macrophages.^71^ These changes result in poor responses to newly encountered antigens and a shift of the immune system toward immunosenescence.

ROS exhibit a hierarchical effect: mild amounts of ROS propagate the reaction of lipid peroxidation chains and induce apoptosis and autophagy in oxidatively damaged cells,^72^ whereas high levels of ROS in cells are considered a driving force for deleterious effects during aging and are tightly associated with inflammaging, aging-related diseases and cancers.^73–75^ Low ROS levels in HSCs and early progenitor cells are essential to maintain their stemness potential compared with that of late differentiated progeny cells.^76^ However, ROS gradually accumulate with the degradation of oxidized proteins by ATP-induced mitochondrial Lon proteases and an impaired mitochondrial oxidized protein repair system.^77^ The increase in ROS gives rise to oxidative stress, and proteasome activity decreases, further leading to substantial protein oxidative modification and functional decline, which lay the foundation for inflammaging and cellular senescence.^78^

### Genetic and epigenetic changes in immunosenescence

Epigenetic mechanisms tightly regulate all biological processes by controlling gene transcription and translation. Several transcription factors (TFs) that are closely related to homeostatic proliferation, activation, and differentiation of T lymphocytes, such as MYC, are expressed at low levels in the thymus of aged rats, which is associated with immunosenescence.^79,80^ Epigenetic dysregulation, including DNA methylation patterns, histone modifications, and noncoding RNAs, is widely identified as a hallmark of aging, increasing autoimmune disorders, and particularly the high tumor risk in elderly individuals. However, the underlying mechanisms responsible for these alterations are not yet known.^81^

#### DNA methylation

As key epigenetic elements that control gene expression, methylation marks are enriched in the promoter region of genes at CpG islands with reduced expression.^82^ Age-related DNA methylation pattern changes, including differential methylation and variable methylation and changes at the level of the global methylome have been identified as the best-characterized epigenetic modifications, suggesting that underlying cellular and molecular changes begin earlier.^83^ and are linked to significant immune cell dysfunction.^84^ DNA methylation patterns that are altered compared with those of healthy individuals have been observed in many age-related diseases. Patients with cerebrovascular disease or atherosclerosis show abnormal DNA methylation patterns in blood or endothelial cells and changes in the DNA methylation patterns of cartilage or bone are observed in patients with osteoarthritis and osteoporosis.^85^ Our current understanding of cumulative changes in DNA methylation over time remains constrained by methods to quantify these changes. Whether it is to find CpG sites that show differences in mean DNA methylation levels or differences in DNA methylation between younger and older individuals, some limitations restrict the study of the mechanism of DNA methylation in aging.

#### Histone modifications

DNA sequences must be accessible to transcription factors and RNA polymerases for genes to be transcribed. The global number of histones defines DNA accessibility.^86^ Simple eukaryotic models show that histone acetylation, phosphorylation, and heterochromatin accumulation increase with aging.^87^ The trimethylation of histone H4 is markedly increased in the liver and kidney of aged rats, leading to transcriptional repression.^88^ Quiescent satellite cells exhibit decreased histone expression and replication-related reduction in histone biosynthesis in aging human fibroblasts.^89^ Heterochromatin is globally lost due to reduced histone synthesis and changes in chromatin structure such as the transition from highly condensed to tightly packed chromatin structures.

In addition, the exchange of histone variants with different primary sequences and properties with classical histones has been observed in aging organisms. Different aging-related studies have evaluated histone variants in mouse, primate, and human cells that include chromatin either coupled to replication or unrelated to replication. The copy-coupling process leads to incorporating new nucleosomes into gaps between preexisting nucleosomes on a genome-wide scale. The addition of replication-independent nucleosomes or subunits occurs locally. Histone variants unrelated to replication can substitute for classical histones and thus potentially alter gene expression programs.^90^ Consequently, the derepression of silenced genes and global gene expression changes may occur.^91^

#### MicroRNAs

As highly conserved single-stranded noncoding RNAs, microRNAs have attracted much attention as epigenetic mediators controlling gene expression at the posttranscriptional level in immunosenescence, particularly in age-related diseases.^28,92^ In the context of immunosenescence, miRNAs mediate chromatin remodeling, release inflammatory mediators, and interfere with the development and differentiation of T and B cells, resulting in decreased immune responses.^93^

### Cellular and molecular level mechanisms in immunosenescence

The mechanisms of immunosenescence underlie an accumulation of damage in various components of the immune system, including the innate immune system and the acquired immune system.^9,16,94^ Innate immune senescence exhibits a decline in antigen processing and presentation capacity and thus a decreased response to stimuli. Characteristics of adaptive immune senescence are a loss of TCR diversity and impaired immunological memory formation. Impaired immune cells with SASP dramatically affect tumor and other age-related disease progression.^1^ Therefore, noting the changes in senescent immune cell subsets is significant.

#### Natural killer cells

Natural killer (NK) cells are characterized by the upregulation of the killer cell immunoglobulin-like receptor (KIR) family and concomitant downregulation of inhibitory natural killer Group 2 member A (NKG2A) receptors from newborns to adults, which appear to be remarkably stable in the majority of elderly individuals.^95^ Only in a minority of subjects was it observed that the age-associated frequency and phenotype of NK cell subsets changed. These elderly donors demonstrated a significant downregulation of cytotoxicity-activating receptors, whose function in NK cells—to promote the antitumor immune response and kill virus-infected cells—is compromised.^96^ However, it is difficult to isolate the impact of immunosenescence from the effect of chronic virus infection, as in most studies, all elderly donors are virus-seropositive.

#### Macrophages

The change in macrophage subpopulation components is intricate. Macrophages can be grouped into proinflammatory, antitumorigenic M1, and anti-inflammatory, protumorigenic M2 subsets.^97^ Compared with young mouse hosts, healthy elderly hepatic tissue and adipose tissue have more M1 macrophages, whereas the immunosuppressive M2 phenotype increases in elderly bone marrow, lymphoid tissues, spleen, muscle, and lung in vivo.^98–100^ Aged M2-like macrophages enhance angiogenesis such that elderly mice are more susceptible to injury-associated angiogenesis, which suggests that aged macrophages play a role in other age-related diseases, including cancers.^101^ The existence of senescent macrophages in vivo remains controversial.^102^ The ability of macrophages to phagocytose pathogens decreases with aging. Compared with young adult donors, macrophages from aged individuals exhibit decreased antigen-presenting capacity due to decreased expression of coreceptors and MHC class II molecules,^103–105^ except for microglia.^106^ Significant downregulation of Toll-like receptor (TLR) expression on macrophages during aging has been described^107^ and is associated with increased Treg cells in aged animals.^108,109^

#### T cells

**Fig. 4:**
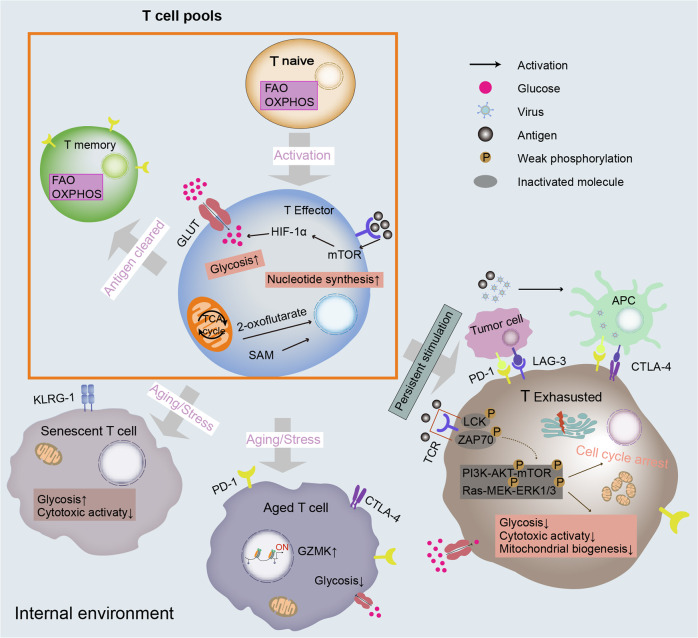
Metabolic and epigenetic modifications for the fate of T cells. Naïve T cells primarily use FAO and OXPHOS to derive their energy. Upon stimulation by antigens, activated mTOR signaling pathways lead to the release of a series of cytokines such as HIF-1α. The effector T cells then exhibit a general increase in glycolytic metabolism and mitochondrial mass with epigenetic reprogramming. Simultaneously, some intermediate metabolites also coordinate with epigenetic remodeling. For example, SAM produced by one-carbon metabolism subsequently promotes nucleotide synthesis. After clearance of the antigen, memory CD8^+^ T cells exhibit a metabolic switch to depressed metabolic activity that utilizes FAO and OXPHOS to meet energy demands. A distinct epigenetic landscape with open chromatin architectures is also displayed on particular loci to maintain longevity. With persistent pathological stimulation, T cells engage in exhausted differentiation with abnormal signals and specific cell cycle-related gene expression, inducing metabolic reprogramming such as decreased aerobic glycolysis, low cytotoxic activity, downregulation of mitochondrial biogenesis, and cell cycle arrest. Finally, aging or stress signals drive all types of T cells to turn into aged/senescent cells. Emerging evidence suggests that aged/senescent T cells also exhibit abnormal metabolic regulation such as mitochondrial dysfunction and a unique epigenetic landscape. APC antigen-presenting cells, CTLA-4 cytotoxic T lymphocyte-associated antigen-4, FAO fatty acid oxidation, GZMK granzyme K, HIF-1α hypoxia-inducible factor-1α, KLRG-1 killer cell lectin-like receptor subfamily G member 1, LAG-3 lymphocyte activation gene 3, MEK mitogen-activated protein kinase kinase, OXPHOS oxidative phosphorylation, PD-1 programmed cell death protein 1

Although the numbers of T cells are more or less constant over the lifespan, various T-cell subpopulations have shown pronounced heterogeneous changes in the context of immunosenescence, which is characterized by a loss in naïve T cells and an increase in highly differentiated CD28^-^ memory T cells or senescent cells.^110^ Emerging evidence suggests that the diversity and output of CD4^+^ T cells are stably maintained through homeostatic mechanisms, but CD8^+^ T cells exhibit significant age-related changes.^9,94^ Focusing on senescent CD8^+^ T cells, the expression of surface molecules changes markedly, although they are not nonexclusive. Most striking is the specific reduction in the costimulatory CD28 family.^111^ Furthermore, high expression of immune biomarkers such as CD57, Tim-3, killer cell lectin-like receptor subfamily G member 1 (KLRG-1), and CD45RA is thought to be implicated in T-cell senescence and has been thoroughly discussed elsewhere.^112–114^ High expression of surface inhibitory receptors in senescent T cells is reminiscent of cellular exhaustion. T-cell exhaustion and aging share a few overlapping functional and phenotypic features with senescence; nevertheless, they have independent regulatory mechanisms and unique developmental signatures (Fig. 4).^115,116^ For example, cell cycle arrest is a hallmark for identifying all types of senescence, but it is not exclusive to senescent cells. Similarly, short-telomere lymphocytes do not proliferate and are resistant to apoptosis but are not metabolically active, whereas senescent T cells is metabolically active.^20,116,117^ Essentially, cellular senescence is irreversible compared with cellular depletion. Regarding T-cell aging, the current knowledge is derived from studies of circulating peripheral blood, which only represents 2% of the T-cell pool.^118^ The understanding of T-cell aging needs to be further deepened. We compare their features in Table 1.

**Table 1:** Comparisons of exhausted, senescent, and aged T cells in metabolic and epigenetic characteristics

Category	Exhaustion	Senescence	Aging	Refs.
Typical feature	proliferative activity ↓ cell cycle arrest: p27, p15 ↑ cyclin E-Cdk2, Cdc25A ↓	proliferative activity ↓ DNA damage ↑ telomere length, telomerase activity ↓ cell cycle arrest: p16, p21, p53↑	T-cell senescence ↑ TCR repertoire ↓ naïve-memory balance↓ effector plasticity↓	^65,68,114,230,231^
Surface markers	PD-1, CTLA-4, Tim-3, LAG-3, BTLA, TIGHT, CD39, CD160, CD244, CD45RO^+^, 4-1BB↑	CD57, KLRG-1, Tim-3, TIGIT, CD45RA, CTLA-4, SA-β-Gal↑ CD27, CD28↓ NKRs↑	PD-1, CTLA-4↑	^5,133,137,232–237^
TCR signaling machinery	LCK, ZAP70↓	LCK, ZAP70, DLG1, Lat, SLP- 76↓	LCK, ZAP70↓	^164^
Cytokine profile	early stage: IL-2↓ intermediate stage: TNFα↓ terminal stage: INF-γ, β-chemokines ↓	SASP, proinflammatory cytokines: IL-6, IL-8, IFN-γ, TNF↑ Inhibitory factors: IL-10, TGF-β ↑ IL-2, IL-7↓	INF-γ, TNF ↓	^133,238^
Epigenetic changes	DNA methylation mediated by Dnmt3a; histone methylation regulated by Jmjd3 and TET	SAHF ↑	global DNA methylation at CpG sites ↓ chromatin accessibility at Gzmk promoters↑	^153,239,240^
Metabolic alterations	glycolysis↓ mitochondrial activity/biogenesis ↓ ROS ↑	glycolysis↑ mitochondrial biogenesis ↓ ROS ↑	glycolysis↓ mitochondrial biogenesis ↓ ROS↑	^56^
Functional alterations	cytotoxic activity↓ effector molecule: GzmB ↓	cytotoxic activity↓ GzmB, perforin↓ suppressive functions↑	cytotoxic activity↓ GzmB ↓ GzmK↑	^69,136,152^

*BTLA* B- and T-lymphocyte attenuator, *Cdc25A* M-phase inducer phosphatase 1, *DLG1* disks large homolog 1, *GzmB* granzyme B, *GzmK* granzyme K, *IL-2* interleukin-2, *IL-6* interleukin-6, *IL-7* interleukin-7, *IL-8* interleukin-8, *IL-10* interleukin-10, *KLRG1* killer cell lectin-like receptor G1, *Lat* linker for activation of T cells, *NKRs* natural killer cell receptors, *SAHF* senescence-associated heterochromatin foci, *SA-β-gal* senescence-associated β-galactosidase

In immunosenescence, metabolic and epigenetic mechanisms play a significant role in determining the fate of T-cell subsets.^5,119,120^ First, pre-TCRs are rearranged in the thymus.^121,122^ The TCR of naïve T cells is stimulated by signals from professional antigen-presenting cells when encountering cognitive antigens, thereby fueling the transition from quiescence to activation of proliferation and differentiation into effector cells.^55,119,123^ A fundamental change among T cells is enhanced aerobic glycolysis in response to increasing expression of glucose transporters (GLUTs) on the cell surface induced by the upregulation of glycolysis-related genes in a c-Myc-dependent manner.^124^ Concomitant with increased glucose metabolism, multiple signaling pathways are integrated to upregulate mitochondrial biogenesis and mtDNA that encodes critical complex components required by oxidative phosphorylation (OXPHOS), but the mechanisms are largely unknown.^125^ Recently, Ron-Harel et al. identified a metabolic signature of mitochondrial proteome remodeling, termed one-carbon metabolism.^123^ Following acute viral infection, naïve T cells rapidly proliferate and differentiate into specialized subpopulations to remove pathogens, kill target cells, and control infection.^119,126^ Most activated T cells die after the antigens are cleared, and a few become memory cells.^127^ Investigations have demonstrated that CD28 synergizes to stimulate the phosphatidylinositol 3-kinase (PI3K)-protein kinase B (PKB/AKT)-mechanism target of the rapamycin (mTOR) signaling axis, which plays a vital role in the rearrangement of TCR and the metabolic reprogramming of glucose, glutamine, and fatty acids during T-cell differentiation.^128–130^ As central transcription regulators, mTOR complex 1 (mTORC1) and mTORC2 demonstrate distinct roles in T-cell differentiation.^131^ Constitutive mTORC1 activation results in the transition from naïve T-cell to CD8^+^ T-cell effector subsets, whereas inhibition of mTORC2 causes the generation of CD8^+^ T-cell memory populations.^132^ During aging, T cells gradually enter a dysfunctional state with body homeostasis disorder and immunosuppression, undergoing metabolic reprogramming and epigenetic remodeling to senescence, aging, and even apoptosis.^133^ TCR rearrangements necessary for generating double-positive thymocytes are impaired because of thymic involution. Changes in CD28 expression in part explain the upregulation of glycolysis in senescent T cells. In T-cell immunosenescence, stress facilitates a failure to express key electron transport chain (ETC) components and encode OXPHOS subunits,^134^ which induces increased ROS generation and decreased proteasome activity, accompanied by decreased levels of mitochondrial synthesis, inefficient one-carbon metabolism, and altered basal lipid metabolism.^52,56,78,135^ Immunocompromised senescent T cells present decreased expression of functional molecules related to cytotoxic activity, such as perforin, granzyme B, and senescence-associated β-galactosidase (SA-β-gal), in vivo.^136,137^

T-cell activation initiates specific gene expression programs that drive cellular differentiation and effector functions associated with changes in the epigenetic landscape.^138^ Activating enhancer-binding protein 4, induced by transient c-Myc gene expression, is a common target for many activation-inducible gene-encoding molecules and is essential for sustained T-cell activation.^139^ Genes associated with effector function are labeled with active epigenetic signatures, thereby facilitating more robust and efficient transcription in response to secondary stimuli, and acquired epigenetic programming can be retained in human CD8^+^ memory T cells for many years, which may account for the durable preservation of CD8^+^ memory T-cell effector potential.^140–142^ During differentiation, methylation marks are acquired at the promoters of genes with reduced expression and lost at the promoters of genes with increased expression.^82^ Using chromatin immunoprecipitation, the codeposition of permissive trimethylation of histone H3 lysine 4 and inhibitory trimethylation of histone H3 lysine 27 at promoter regions is recognized as a hallmark of naïve CD8^+^ T-cell differentiation.^143^ The number of methylation and methylome changes significantly differs among T-cell subsets, particularly in CD8^+^ T cells with aging. Altered global DNA methylation patterns have recently been identified as one of the best-characterized epigenetic modifications in T-cell aging.^83^ CpG islands of silent genes were hypermethylated and enriched for repressive histone marks, and the majority of age-related hypomethylated sites were located at DNA regions flanking CpG islands. Tserel et al. found strong negative correlations between methylation and expression levels of genes associated with cellular immune response and differentiation (e.g., CD27 and SATB1) in CD8^+^ T-cell subsets, which suggests a link between age-related epigenetic changes and impaired T-cell function. Intriguingly, the degree of DNA methylation at specific CpG sites reflects the organic aging rate in several studies.^144^ In various aging models, a decline in core histone protein expression levels has been observed.^145^ Typically, senescent T cells exhibit unique chromatin condensation, termed senescence-associated heterochromatic foci (SAHF); a global increase in chromatin accessibility; and a global loss of linker histone H1.^146,147^ Hypomethylation coinciding with changes in histone modifications, particularly repetitive genomic sequences in heterochromatin, may instigate genomic instability and premature senescence.^148,149^ The expression level of miR-92a in CD8^+^ T cells declines significantly with age and is associated with decreased naïve T cells in immunosenescence.^150^ Overexpression of miR-24 in senescent T cells with downregulated CD28 expression caused the expression of histone variant H2AX to decline, thereby reducing the repair ability of T cells for the DNA damage response.^151^ These age-dependent epigenetic changes also arise in immune-specific genomes, leading to T-cell senescence. Interestingly, available assays revealed that the dominant aged signatures of T cells resemble the hallmarks of exhausted T cells. Comprehensive profiling of immune tissue across multiple mouse organs found a subset of clonal age-associated granzyme K (GZMK)^-^expressing CD8^+^ T cells with exhausted-like phenotypes.^152^ During aging, increasing GZMK^+^ CD8^+^ effector memory cells from human peripheral blood mononuclear cells (PBMCs) shared specific alterations of the epigenetic landscape and exhausted expression marks with mouse age-associated GZMK-expressing CD8^+^ T cells.^152^ Basic leucine zipper ATF-like TF, which is extensively expressed in aged CD8^+^ T cells, plays a prominent role in T-cell exhaustion differentiation.^153^ Terminally exhausted T cells typically exhibit upregulation of chromatin accessibility at the promoters of effector genes associated with granzyme B (GZMB) and interferon-γ (IFN-γ), which did not change with age, suggesting that the mechanisms of aging and T-cell exhaustion progression are merely similar in part.^153^ Although intensive studies of T cells have provided some insight into immunosenescence, they still have several limitations and need to be considered in future investigations.

#### B cells

Plasma cells derived from B cells are the sole producer of lasting protective antibodies, developing an immunological memory after infection or vaccination.^154^ Immunosenescence results in the remodeling of the B-cell compartment.^155^ The E47 mRNA degradation rate is significantly increased in B cells from elderly mice.^156^ The reduced output of B cells, combined with diminished expression of the autoimmune regulator and autoantigen genes in thymic B cells, drastically hinders the development of humoral immunity in response to infectious pathogens in the elderly.^157,158^

#### Other cells

According to recent research, neutrophils also modulate T-cell function, and their function is regulated with aging. As central orchestrators of the immune response, the critical role of dendritic cells (DCs) in the maintenance of tolerance, antigen presentation to T cells, and endocytosis is also reduced in aged individuals; however, there is no significant effect on the numbers and phenotype of DCs in aged subjects.^159^ Aged HSCs show more substantial myeloid lineage potential. The expansion of myeloid-derived suppressor cells (MDSCs) promotes immunosenescence and induces severe bystander effects in host tissues by secreting inflammatory factor transforming growth factors (TGF-β) and IL-10.^160^

### Inflammaging and its role in positive feedback in immunosenescence

**Fig. 5:**
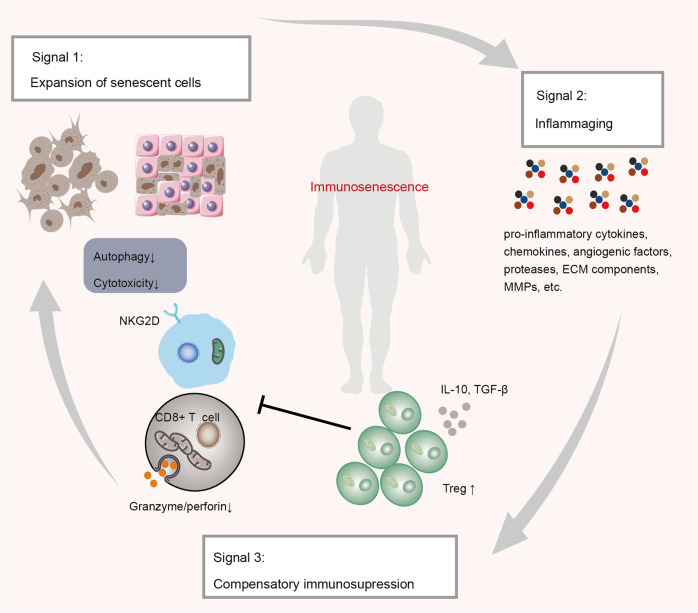
The circuit between cellular senescence and inflammaging promotes immunosenescence and tumorigenesis. Senescent cells gradually accumulate in vivo. Consequently, the increased SASP of senescent cells aggravates the inflammatory state, which activates counteracting immunosuppression. Subsequently, suppressive Treg cells secrete inhibitory cytokines, such as IL-10 and TGF-β, to prevent CD8 + T cells from surveilling and clearing senescent cells. In addition, senescent cells can enable sustained activation of the NKG2D receptor by releasing soluble NKG2D ligands from their cell surface or by increasing the expression of specific inhibitory proteins. Sustained activation of NKG2D receptor function is inhibited, thereby impairing immune system function and enhancing the expansion of senescent cells in the TME. ECM extracellular matrix, MMPs matrix metalloproteinases, NKG2D natural killer Group 2 member D

Immunosenescence involves inflammaging and progressive deposition of senescent cells.^161,162^ Inflammation and cellular senescence are not negative phenomena. However, SASP cells secrete a plethora of soluble molecules, typically including proteases, fibroblast growth factor 2 and hepatocyte growth factor, chemokines, angiogenic factors, proinflammatory cytokines, matrix metalloproteinases (MMPs), and extracellular matrix (ECM) components.^23,163^ Active p-p38 mitogen-activated protein kinase (MAPK) in senescent T cells inhibits autophagy protein nine in an mTOR-independent manner, thus restraining autophagy.^56^ In addition, senescent T cells with programmed cell death protein 1 (PD-1) can affect the phosphorylation of zeta-chain-associated protein kinase 70 (ZAP70) induced by lymphocyte-specific protein tyrosine kinase (LCK) to antagonize TCR signals directly, thereby further activating the P38 pathway and inhibiting PI3K-AKT-mTOR signaling, ultimately causing autophagy loss (Fig. 4).^164^ Failure of autophagy leads to the accumulation of T cells with the SASP phenotype, which in turn induces mitochondrial dysfunction, stimulates the generation of myeloid-derived suppressor cells, increases ROS levels, and exacerbates inflammaging.^56,162^ Mitochondrial metabolic disorders cause a type 1 cytokine inflammatory storm of TNF-α and IFN-γ, subsequently inducing inflammaging in peripheral tissues.^2,165^ Conceivably, sustained secretion of the SASP factor in tissues is a significant source of inflammaging^166–168^ (Fig. 5). Age-regulated tissue-specific macrophages and neutrophils may cause immunosuppressive chronic low-grade inflammation, resulting in many diseases. Increased TNF-α levels were associated with reduced CD28 expression and defective T-cell responses.^59^ Interestingly, autocrine and bystander effects were observed whereby SASP induced senescence propagation, thereby causing inflammaging and several senescence-related diseases, in particular, diverse malignancies.^169^ There appears to be a positive feedback regulatory circuit between inflammaging and cellular senescence, which impairs the immune surveillance and autophagy of senescent cells, thus allowing premature immunosenescence. Whether early modulation of inflammaging can prevent or delay cellular senescence, immunosenescence, and age-related diseases needs to be tested in clinical trials.

## Multiple diseases associated with immunosenescence

Older adults become more susceptible to multiple diseases with the advance of immunosenescence. It is essential to study the interplay and mechanisms to bring viable solutions for preventing and treating these diseases and increasing the health span of elderly populations.

### Cardiovascular disease

Inflammation increases proinflammatory cytokine levels and the likelihood of endothelial injury, vascular remodeling damage, and atherosclerosis. Inflammaging conditions recruit monocytes and trigger them to convert into lipid-containing, senescent foamy macrophages, which then accumulate and accelerate atherosclerotic progression. Foamy macrophages with SA-β-gal accumulate in fatty streaks of mice. They upregulate the SASP factors MMP12 and MMP13 and recruit monocytes, stimulating more conversion to foamy macrophages and promoting plaque instability.^170^ In patients infected with cytomegalovirus, CD8^+^CD28^−^ T-cell expansion is a risk factor for vascular dysfunction and is strongly associated with suffering from atherosclerosis and acute coronary syndrome.^171^ Recently, Yu showed that high frequencies of senescent CD8^+^CD57^+^ T cells present in the PBMCs of patients with acute myocardial infarction have proinflammatory and tissue-homing properties and are linked to short-term cardiovascular mortality in patients.^172^

### Autoimmune disease

Aging is associated with an increased incidence of autoimmune diseases that predominantly affect women. Rheumatoid arthritis (RA) is a chronic inflammatory disease associated with symmetrical and destructive inflammation in joints and other tissues. As autoimmune diseases progress, there is a higher prevalence of age-related complications. The most common comorbidities in RA may include osteoporosis, cancer, cardiovascular disease, and neuropsychiatric disorders.^173^ Patients with RA develop features of premature immunosenescence such as decreased thymic function, expanded late-differentiated effector T cells, increased telomeric attrition, and a proinflammatory phenotype.^174^ Interestingly, RA patients presenting with extra-articular manifestations also have an increased frequency of aged T cells.^175^ These CD28^−^ T-cell subsets may contribute to inflammaging and further aggravate the disease severity of RA upon stimulation by increasing the production of inflammatory factors.^176,177^ Favorable clinical responses were identified using specific treatments significantly reducing CD8^+^ CD28^−^ T cells in RA patients.^178^ Increased telomere erosion, resulting in increased autoreactivity with self-antigens and loss of immune tolerance, is associated with the development of atherosclerotic plaques and cardiovascular disease in RA, all of which indicate potential age-related risk factors for RA development.^179^ Thus, immunosenescence may be a prerequisite for the increased incidence of autoimmune diseases.

### Neurodegenerative diseases

Immunosenescence has been repeatedly implicated in cognitive processes and neurodegenerative diseases, which place a tremendous economic and social burden on society. For example, levels of proinflammatory factors such as IL-6 are associated with depression, fatigue, and cognitive impairment and are elevated in Alzheimer’s and Parkinson’s patients.^50,180^ Accelerated epigenetic aging has also been shown to be associated with the development of neurodegenerative diseases.^181^ Astrocytes in aged brains also express more significant levels of p16 than those in young brains.^182^ Cellular senescence also prompts a physiological aging shift into neurodegeneration.^183^ Alzheimer’s disease (AD) is a distinct neurodegenerative disease associated with immunosenescence. The silencing transcription factor REST protects neurons from oxidative stress and β-amyloid toxicity. Physiologically aged human brains showed higher REST mRNA and protein levels than AD patients. Targeted deletion of REST in the mouse brain promotes age-related neurodegeneration. The expression of transgenic human Rest in *C*. *elegans* demonstrated the neuroprotective effect of REST in reducing sensitivity to oxidative stress.^184,185^ However, published evidence on whether causal links exist between immunosenescence and neurodegeneration is still limited.

### Cancers

Investigations have discovered that several hallmarks of immunosenescence such as epigenetic modifications, mitochondrial dysfunction, and cellular senescence may account for cancer features in older adults.^1^ Tumor response and tumor aggressiveness differ between young and older adults.^16,186^ Age is associated with more aggressive tumor phenotypes in many tumor types. Gomes et al. emphasized that methylmalonic acid upregulation in the serum of older adults induces SOX4 expression, which triggers transcriptional reprogramming, further endowing cancer cells with greater aggressiveness.^187^ However, although the overall incidence remains higher for some cancers in older patients, cancers in younger patients are more aggressive and have poorer outcomes. Compared with young melanoma patients, ECM changes in elderly patients result in lower sentinel lymph node metastasis rates but higher mortality.^188^ Studies have suggested that slower tumor growth and metastasis in elderly patients with bronchial cancer are due to senescent host factors hindering the growth and spread of invasive tumors.^189^ Another retrospective study of 1869 women with breast cancer found less aggressive features in elderly patients.^190^ These findings suggest that immunosenescence is closely linked with tumorigenesis and progression.

### Coronavirus disease 2019

Coronavirus disease 2019 (COVID-19), first reported in December 2019, has spread worldwide at an unprecedented rate with profound and ongoing health and socioeconomic impacts. The pathogen of COVID-19 is a highly contagious respiratory β-coronavirus, severe acute respiratory syndrome coronavirus 2 (SARS-CoV-2).^191^ Highly heterogeneous COVID-19 can manifest as asymptomatic, mild, severe, and even lethal because of the host’s underlying complications and other factors. Elderly individuals and adults with inflammatory conditions are at heightened risk for developing and dying from COVID-19.^192^ Age-related decline and dysregulation of immune function, i.e., immunosenescence, may play an important role in older adults being more susceptible to severe outcomes with COVID-19. A clinical trial (NCT04510194) is ongoing to investigate whether anti-senescence and reduced age-related immune decline could improve nonhospitalized COVID-19 outcomes. There remains much to be learned about immune responses to SARS-CoV-2 infection.

## Potential therapeutic strategies to improve responses among older adults

### Selective elimination of senescent cells and related clinical trials

T-cell senescence has been recognized as one of the causative factors of aging and immunosenescence. The production and expansion of senescent cells synergistically with immunosenescence depresses the efficacy of adoptive cell transfer therapies.^193^ Senescence is not an acute transition but is progressive and phased from a temporary or reversible state to a chronic irreversible state.^163,194^ Simple anti-senescence may not produce the expected therapeutic effects and can harm healthy tissues in elderly individuals. Recently, several findings have challenged the irreversibility of cellular senescence.^195^ The derepression of the human telomerase reverse transcriptase gene restores telomerase activity, allowing oncogene-induced senescent cells to re-enter the cell cycle. Senescent cells that regain stemness acquire increased proliferative capacity and tumorigenesis potential.^167,196^ In summary, the role of senescent T cells in the growth, invasion, and resistance to therapies for immunosenescence and age-related diseases remains questionable.

Several small molecules have exhibited promising activity against cellular senescence. For example, rapamycin was observed to reverse the increased SASP of senescent cells and increase antiviral gene expression in elderly individuals.^197^ However, these agents lack selectivity and may produce substantial side effects such as inflammatory responses and even deterioration of several normal tissues; thus, they do not appear be the optimal treatment choice.^198^ Immunotherapy, such as chimeric antigen receptor-T-cell (CAR-T) and TCR therapy, has shown revolutionary clinical benefits in patients with advanced or conventional therapy-resistant cancers.^199,200^ However, the effect of immune checkpoint inhibitors, which can suppress the expression of specific proteins produced by senescent cells or prevent inhibitory receptor activation, is controversial because of the low participation of aged patients in clinical trials.^201^ Recently, several researchers discovered that patients with non-small cell lung cancer are resistant to immune checkpoint-blocking treatments.^202^ According to preclinical research, immunosenescence is mainly responsible for the response to therapy in 8-week-old mice, which may explain, in part, why many successful preclinical therapies fail in clinical trials. We propose that CAR-T cells targeting senescence-specific surface antigens may be a plausible therapeutic perspective. We have collected several recent clinical immunotherapy trials on CAR-T cells or targeting senescence-associated biomarkers and collated them into Table 2. Unfortunately, most clinical trials of the characteristic biomarkers CD57 and KLRG-1 of senescent T cells have not been applied. Recent research has found an emerging cell surface protein widely induced during senescence: urokinase-type plasminogen activator receptor (uPAR). uPAR-specific CAR T cells could effectively eliminate senescent cells in mice, although further investigations are required to explore whether they have the necessary safety profile for clinical application.^203^ Despite this caveat, there is growing interest in identifying novel markers of senescence that may also have prognostic potential.

**Table 2:** List of the current immunotherapy targeting senescence-associated biomarkers

Conditions	interventions	Target	Phase	Identifier	Status
HIV infection associated tumors	Ipilimumab and Nivolumab	PD-1, CTLA-4	Phase 1	NCT02408861	Recruiting
Advanced solid tumor malignancies	Sym023	Tim-3	Phase 1	NCT03489343	Completed
Solid tumor, lymphoma	LB1410	Tim-3, PD-1	Phase 1	NCT05357651	Not yet recruiting
Melanoma	TSR-022, TSR-042	Tim-3, PD-1	Phase 2	NCT04139902	Recruiting
Liver cancer	TSR-022, TSR-042	Tim-3, PD-1	Phase 2	NCT03680508	Recruiting
Advanced cancer	COM902	TIGIT	Phase 1	NCT04354246	Recruiting
Locally advanced cancer or metastatic cancer	OMP-313M32	TIGIT	Phase 1	NCT03119428	Terminated
Relapsed refractory multiple myeloma	pomalidomide, dexamethasone	TIGIT, LAG-3	Phase 2	NCT04150965	Recruiting
Cervical cancer	Ociperlimab	TIGIT, PD-1	Phase 2	NCT04693234	Active
Non-small-cell lung carcinoma	AZD2936	TIGIT, PD-1	Phase 2	NCT04995523	Recruiting
Esophageal squamous cell carcinoma	Tislelizumab, Ociperlimab	TIGIT, PD-1	Phase 2	NCT04732494	Recruiting
Multiple myeloma	CD3/CD28	KLRG-1	Phase 2	NCT01426828	Completed

*CAR* chimeric antigen receptor, *CMV* cytomegalovirus

### Targeting signal transduction improves immunosuppression and enhances immune function

At present, some assays for senescent cells have achieved beneficial results. Removal of p16Ink4a-positive senescent cells extends life expectancy in mice, slowing SASP emergence and age-associated functional deterioration of organs and tissues.^204,205^ Based on our previous discussion, we propose a concept that regulates the metabolic pathways associated with T-cell senescence mentioned above to prevent premature T-cell senescence and enhance immune function. Treg cells exhibit increased glucose and glycolytic metabolism and subsequently induce cellular senescence and an immunosuppressive microenvironment.^26^ An increased proportion of Tregs is a significant obstacle to a successful immune response in age-related diseases.^65^ It has been indicated that Treg-induced T-cell senescence can be blocked by regulating glucose metabolism in vitro and in vivo in animal models.^63,206^ Mechanistically, poly-G3 activated human TLR8 in Treg cells and then dramatically drove the downregulation of GLUT1 as well as the GLUT3 gene and glucose metabolism-associated enzyme genes such as hexokinase and phosphofructokinase, promoting the transfer of GLUT1 and GLUT3 from the cell membrane surface to intracellular storage sites and subsequently inhibiting glucose uptake, transport, and glycolysis. In addition, activation of TLR8 signaling downregulated the levels of cyclic adenosine monophosphate and mTORC1-HIF-1α signaling, inhibiting tumor cell metabolism and senescence. Recent studies have shown that senescent T cells can regain function through the MAPK pathway.^68^ Additionally, AMPK signaling has acted as a novel therapy to inhibit cellular senescence and improve the aging immune system.^207^ Based on previous reviews, whether anti-senescence and improved age-related immune decline could improve nonhospitalized COVID-19 outcomes is currently being investigated in an ongoing clinical trial (NCT04510194). The extent to which senescent cells accumulate in humans correlates with inflammation and organ damage, and it is essential to investigate whether plasma protein profiles associated with senescent cell burden can be developed.

Intermediates of metabolic pathways, such as substrates and cofactors, are essential for numerous chromatin- and DNA-modifying enzymes; therefore, metabolic changes in dysfunctional T cells within immunosenescence could touch off epigenetic reprogramming. For example, downregulated glycolytic levels in exhausted and aged T cells can influence the availability of NAD^+^ and NADH downstream in aged-like T cells, thereby affecting sirtuin-2-mediated histone deacetylation and resulting in reduced chromatin accessibility.^208^ Additionally, the low production of S-adenosylemethionine (SAM) due to defective one-carbon metabolism in senescent T cells led to impaired DNA and histone methylation.^126^ Similarly, examples of epigenetic modulation mediating T-cell metabolism are frequently discussed in the context of immunosenescence. The decline in miR-181a is thought to be an example of antagonistic pleiotropy; it is beneficial for T-cell development in young adults and prevents autoimmune diseases, but the decreased expression of miR-181a during aging leads to its diminished inhibitory effect, and the expression of dual-specific phosphatase DUSP6, constitutively expressed in T-cell cytoplasm, is upregulated, causing defects in the ERK signaling pathway, which impairs both positive and negative selection and TCR sensitivity.^209,210^ Moreover, aged CD8^+^ T cells, irrespective of their differentiation state, displayed a loss of chromatin accessibility at specific promoters and consequently caused decreased levels of nuclear respiratory factor 1 binding and ETC complexes, which likely resulted in metabolic dysfunction facilitating immunosenescence.^211^ However, it remains unclear whether these alterations lead to T-cell senescence or whether T-cell senescence leads to metabolic and epigenetic reprogramming.

Given the regulatory nature of metabolism and the partial reversibility of epigenetic mechanisms, we propose a potential hypothetical driving axis that targets epigenetic alterations and metabolic reprogramming interacting in T-cell senescence against immunosenescence. Because tricarboxylic acid cycle intermediates such as fumarate, succinate, and α-ketoglutarate have been identified as vital epigenetic regulators, studies targeting mitochondria and their metabolites are considered critical to integrate metabolism with epigenetic modifications.^212^ Apart from bioenergetic and biosynthetic functions, mitochondria act as signaling organelles whose fitness is closely linked to nuclear activity, triggering epigenetic programs to adapt to homeostatic stress during aging. Mitochondrial dysfunction has been observed among exhausted, senescent, and aging T cells.^2,116,135^ In mice, T lymphocytes with mitochondrial transcription factor A (TFAM) deficiency cause multiple aging-related metabolic and cognitive changes, accelerating premature death.^2^ Consistent with these results, CD4^+^ and CD8^+^ T cells with inherent differences in mitochondrial content showed discrete susceptibility to senescence and aging.^213^ In this sense, deeper links between metabolic and epigenetic changes must be explored to find more promising treatment strategies. Desdin-Mico et al. confirmed that using nicotinamide adenine dinucleotide precursors partially rescues premature senescence in mice that is mediated by TFAM-deficient T cells.^2^ Evidence from multiple trials suggests that restoration of mitochondria or mitochondrial metabolites via the epigenome may be a promising direction for improving impaired T-cell immunity and immune system disorders. A series of physiological factors such as diet, exercise, enteric microorganisms, and the circadian clock have been well defined as potential regulators that prevent T-cell senescence and alleviate the immunosuppressive milieu via metabolic and epigenetic programs, thereby enhancing immune function and delaying the initiation and development of cancers.^115,214–222^ In this sense, deeper links between metabolic and epigenetic changes must be explored to find more promising therapeutic strategies.

## Vaccination in the elderly

Adults over 60 years old have increased susceptibility to infectious diseases, which severely reduces vaccination effectiveness. Using a systems biology approach to identify early gene signatures is pioneering work for predicting immune responses in humans.^223^ Pulendran and colleagues conducted a series of clinical studies over three years to evaluate the responses to influenza vaccination in adults.^224^ According to previous studies, later antibody titers correlate with early molecular signatures and can be accurately predicted. An analysis of system-wide methylation levels identified groups of CpG islands that affected the expression of several genes with established roles in humoral immunity.^225^ A high proportion of senescent T cells has already been identified to be associated with reduced vaccine efficacy in the elderly.^47,226^ Extensive analysis of the elderly has suggested that humoral responses to influenza vaccination were significantly associated with age-related changes in T-cell populations and function.^227^ Genetic signatures associated with B-cell proliferation, identified by gene set enrichment analysis, can accurately predict high and low responders to influenza vaccines.^228^ Alterations in the gut microbiome mediate signals that provide differential antibody responses to peripheral immune cells.^229^ These findings allow for identification of the underlying mechanism of the vaccine response. Multiple factors can affect immunosenescence and be affected by immunosenescence, further confirming that immunosenescence makes vaccines less potent through specific signaling pathways.

## Conclusions and further perspectives

Overall, the function and fitness of the immune system are critical factors for organism homeostasis, resistance against antigens, and successful immunotherapy. Metabolism connected with epigenetic pathways strongly drives immune system aging and cellular senescence. Understanding of the molecular mechanisms related to immunosenescence remains limited, and gold-standard biomarkers for senescence remain lacking. The data suggest synergy between antiaging treatments and checkpoint immunotherapy. For example, rapamycin, combined with immune therapy, achieved better therapeutic effects by interfering with SASP, whereas successful immune checkpoint inhibitor therapy in the elderly remains limited, and many therapies are still in preclinical or clinical trials. Therefore, systematic studies on immunosenescence are needed. Decreased T-cell output induced by thymic degeneration in young adults can be reversed by IL-7 supplementation, whereas thymic rejuvenation in older cohorts does not restore T-cell receptor diversity. Severe reversal of the senescent cellular state confers increased proliferative capacity and tumorigenic potential to cells reentering the cell cycle. Although significant advances have been made in clinical and basic immunosenescence research, many results are based on mouse models. The existing immunological techniques and experimental progress must fully reveal the complexities of the human immune system. There is a need to understand the more profound effects of immunosenescence on age-related diseases, particularly in terms of tumor progression, and develop new scientific approaches to establish more convincing in vivo models of aging to search for potential mechanisms that induce immune suppression, which may provide emerging insights into antiaging immunity and antitumor therapy.

## References

[1] Fane M, Weeraratna A (2020). How the ageing microenvironment influences tumour progression. Nat. Rev. Cancer.

[2] Desdin-Mico G (2020). T cells with dysfunctional mitochondria induce multimorbidity and premature senescence. Science.

[3] Finn OJ (2012). Immuno-oncology: understanding the function and dysfunction of the immune system in cancer. Ann. Oncol..

[4] Candeias SM, Gaipl US (2016). The immune system in cancer prevention, development and therapy. Anticancer Agents Med. Chem..

[5] Lian J, Yue Y, Yu W, Zhang Y (2020). Immunosenescence: a key player in cancer development. J. Hematol. Oncol..

[6] Alizadeh D (2019). IL15 enhances CAR-T cell antitumor activity by reducing mTORC1 activity and preserving their stem cell memory phenotype. Cancer Immunol. Res..

[7] Walford RL (1964). The immunologic theory of aging. Gerontologist.

[8] Pawelec G (2007). Immunosenescence comes of age. Symposium on aging research in immunology: the impact of genomics. EMBO Rep..

[9] Thomas R, Wang W, Su DM (2020). Contributions of age-related thymic involution to immunosenescence and inflammaging. Immun. Ageing.

[10] McElhaney JE, Effros RB (2009). Immunosenescence: what does it mean to health outcomes in older adults?. Curr. Opin. Immunol..

[11] Kennedy BK (2014). Geroscience: linking aging to chronic disease. Cell.

[12] Franceschi C (2000). Inflamm-aging. An evolutionary perspective on immunosenescence. Ann. N. Y. Acad. Sci..

[13] George AJ, Ritter MA (1996). Thymic involution with ageing: obsolescence or good housekeeping?. Immunol. Today.

[14] Solana R (2012). Innate immunosenescence: effect of aging on cells and receptors of the innate immune system in humans. Semin. Immunol..

[15] Pang WW (2011). Human bone marrow hematopoietic stem cells are increased in frequency and myeloid-biased with age. Proc. Natl Acad. Sci. USA.

[16] Palmer S, Albergante L, Blackburn C, Newman T (2018). Thymic involution and rising disease incidence with age. Proc. Natl Acad. Sci. USA.

[17] Whiteside TL (2008). The tumor microenvironment and its role in promoting tumor growth. Oncogene.

[18] Baitsch L (2012). The three main stumbling blocks for anticancer T cells. Trends Immunol..

[19] Su DM, Aw D, Palmer DB (2013). Immunosenescence: a product of the environment?. Curr. Opin. Immunol..

[20] Crespo J (2013). T cell anergy, exhaustion, senescence, and stemness in the tumor microenvironment. Curr. Opin. Immunol..

[21] Akbar AN, Henson SM, Lanna A (2016). Senescence of T lymphocytes: implications for enhancing human immunity. Trends Immunol..

[22] Hayflick L, Moorhead PS (1961). The serial cultivation of human diploid cell strains. Exp. Cell Res..

[23] Gorgoulis V (2019). Cellular senescence: defining a path forward. Cell.

[24] Campisi J, d’Adda di Fagagna F (2007). Cellular senescence: when bad things happen to good cells. Nat. Rev. Mol. Cell Biol..

[25] Courtois-Cox S, Jones SL, Cichowski K (2008). Many roads lead to oncogene-induced senescence. Oncogene.

[26] Yousefzadeh M (2021). An aged immune system drives senescence and ageing of solid organs. Nature.

[27] Ovadya Y (2018). Impaired immune surveillance accelerates accumulation of senescent cells and aging. Nat. Commun..

[28] Lyu G (2018). TGF-beta signaling alters H4K20me3 status via miR-29 and contributes to cellular senescence and cardiac aging. Nat. Commun..

[29] Dimri GP (1995). A biomarker that identifies senescent human cells in culture and in aging skin in vivo. Proc. Natl Acad. Sci. USA.

[30] Sharpless NE, Sherr CJ (2015). Forging a signature of in vivo senescence. Nat. Rev. Cancer.

[31] Accardi G, Caruso C (2018). Immune-inflammatory responses in the elderly: an update. Immun. Ageing.

[32] Nikolich-Zugich J (2018). The twilight of immunity: emerging concepts in aging of the immune system. Nat. Immunol..

[33] Lanna A (2017). A sestrin-dependent Erk-Jnk-p38 MAPK activation complex inhibits immunity during aging. Nat. Immunol..

[34] Ucar D (2017). The chromatin accessibility signature of human immune aging stems from CD8(+) T cells. J. Exp. Med..

[35] Fulop T (2014). On the immunological theory of aging. Interdiscip. Top. Gerontol..

[36] Palmer DB (2013). The effect of age on thymic function. Front. Immunol..

[37] Goronzy J, Weyand C (2013). Understanding immunosenescence to improve responses to vaccines. Nat. Immunol..

[38] Pawelec G (2012). Hallmarks of human “immunosenescence”: adaptation or dysregulation?. Immun. Ageing..

[39] Flores KG (1999). Analysis of the human thymic perivascular space during aging. J. Clin. Invest..

[40] Lynch HE (2009). Thymic involution and immune reconstitution. Trends Immunol..

[41] Wertheimer AM (2014). Aging and cytomegalovirus infection differentially and jointly affect distinct circulating T cell subsets in humans. J. Immunol..

[42] Sauce D (2009). Evidence of premature immune aging in patients thymectomized during early childhood. J. Clin. Invest..

[43] Qi Q, Zhang DW, Weyand CM, Goronzy JJ (2014). Mechanisms shaping the naive T cell repertoire in the elderly - thymic involution or peripheral homeostatic proliferation?. Exp. Gerontol..

[44] Hale JS, Boursalian TE, Turk GL, Fink PJ (2006). Thymic output in aged mice. Proc. Natl Acad. Sci. USA.

[45] Fulop T (2017). Immunosenescence and inflamm-aging as two sides of the same coin: friends or foes?. Front. Immunol..

[46] Feldman N, Rotter-Maskowitz A, Okun E (2015). DAMPs as mediators of sterile inflammation in aging-related pathologies. Ageing Res. Rev..

[47] Effros RB, Dagarag M, Spaulding C, Man J (2005). The role of CD8+ T-cell replicative senescence in human aging. Immunol. Rev..

[48] Bruunsgaard H (2003). Elevated levels of tumor necrosis factor alpha and mortality in centenarians. Am. J. Med..

[49] Ershler WB, Keller ET (2000). Age-associated increased interleukin-6 gene expression, late-life diseases, and frailty. Annu. Rev. Med..

[50] Puzianowska-Kuznicka M (2016). Interleukin-6 and C-reactive protein, successful aging, and mortality: the PolSenior study. Immun. Ageing.

[51] Ferrucci L, Fabbri E (2018). Inflammageing: chronic inflammation in ageing, cardiovascular disease, and frailty. Nat. Rev. Cardiol..

[52] Calcinotto A (2019). Cellular senescence: aging, cancer, and injury. Physiol. Rev..

[53] Campisi J (2013). Aging, cellular senescence, and cancer. Annu. Rev. Physiol..

[54] McHugh D, Gil J (2018). Senescence and aging: causes, consequences, and therapeutic avenues. J. Cell Biol..

[55] Patsoukis N (2015). PD-1 alters T-cell metabolic reprogramming by inhibiting glycolysis and promoting lipolysis and fatty acid oxidation. Nat. Commun..

[56] Henson SM (2014). p38 signaling inhibits mTORC1-independent autophagy in senescent human CD8(+) T cells. J. Clin. Invest..

[57] Doran MF (2002). Trends in incidence and mortality in rheumatoid arthritis in Rochester, Minnesota, over a forty-year period. Arthritis Rheum..

[58] Jemal A (2007). Cancer statistics, 2007. CA Cancer J. Clin..

[59] Ponnappan S, Ponnappan U (2011). Aging and immune function: molecular mechanisms to interventions. Antioxid. Redox Signal.

[60] Nikolich-Zugich J (2008). Ageing and life-long maintenance of T-cell subsets in the face of latent persistent infections. Nat. Rev. Immunol..

[61] Khan N (2002). Cytomegalovirus seropositivity drives the CD8 T cell repertoire toward greater clonality in healthy elderly individuals. J. Immunol..

[62] Cristofalo VJ (2004). Replicative senescence: a critical review. Mech. Ageing Dev..

[63] Ye J (2012). Human regulatory T cells induce T-lymphocyte senescence. Blood.

[64] Ye J (2013). Tumor-derived gammadelta regulatory T cells suppress innate and adaptive immunity through the induction of immunosenescence. J. Immunol..

[65] Liu X (2018). Regulatory T cells trigger effector T cell DNA damage and senescence caused by metabolic competition. Nat. Commun..

[66] Sitkovsky MV, Kjaergaard J, Lukashev D, Ohta A (2008). Hypoxia-adenosinergic immunosuppression: tumor protection by T regulatory cells and cancerous tissue hypoxia. Clin. Cancer Res..

[67] Vang T (2001). Activation of the COOH-terminal Src kinase (Csk) by cAMP-dependent protein kinase inhibits signaling through the T cell receptor. J. Exp. Med..

[68] Lanna A, Henson SM, Escors D, Akbar AN (2014). The kinase p38 activated by the metabolic regulator AMPK and scaffold TAB1 drives the senescence of human T cells. Nat. Immunol..

[69] Jiang Y, Li Y, Zhu B (2015). T-cell exhaustion in the tumor microenvironment. Cell Death Dis..

[70] Almanzar G (2005). Long-term cytomegalovirus infection leads to significant changes in the composition of the CD8+ T-cell repertoire, which may be the basis for an imbalance in the cytokine production profile in elderly persons. J. Virol..

[71] Wang D (2019). Macrophage-derived CCL22 promotes an immunosuppressive tumor microenvironment via IL-8 in malignant pleural effusion. Cancer Lett..

[72] Su LJ (2019). Reactive oxygen species-induced lipid peroxidation in apoptosis, autophagy, and ferroptosis. Oxid. Med. Cell Longev..

[73] Harman D (2003). The free radical theory of aging. Antioxid. Redox Signal..

[74] Finkel T, Holbrook NJ (2000). Oxidants, oxidative stress and the biology of ageing. Nature.

[75] Licastro F (2005). Innate immunity and inflammation in ageing: a key for understanding age-related diseases. Immun. Ageing.

[76] Diehn M (2009). Association of reactive oxygen species levels and radioresistance in cancer stem cells. Nature.

[77] Ugarte N, Petropoulos I, Friguet B (2010). Oxidized mitochondrial protein degradation and repair in aging and oxidative stress. Antioxid. Redox Signal..

[78] Das R, Ponnappan S, Ponnappan U (2007). Redox regulation of the proteasome in T lymphocytes during aging. Free Radic. Biol. Med..

[79] Sidler C (2013). Immunosenescence is associated with altered gene expression and epigenetic regulation in primary and secondary immune organs. Front. Genet..

[80] Angelosanto JM, Wherry EJ (2010). Transcription factor regulation of CD8+ T-cell memory and exhaustion. Immunol. Rev..

[81] Sen P, Shah PP, Nativio R, Berger SL (2016). Epigenetic mechanisms of longevity and aging. Cell.

[82] Henning AN, Roychoudhuri R, Restifo NP (2018). Epigenetic control of CD8(+) T cell differentiation. Nat. Rev. Immunol..

[83] Tserel L (2015). Age-related profiling of DNA methylation in CD8+ T cells reveals changes in immune response and transcriptional regulator genes. Sci. Rep..

[84] Bocker MT (2011). Genome-wide promoter DNA methylation dynamics of human hematopoietic progenitor cells during differentiation and aging. Blood.

[85] Seale K (2022). Making sense of the ageing methylome. Nat. Rev. Genet..

[86] Zhang W, Qu J, Liu GH, Belmonte JCI (2020). The ageing epigenome and its rejuvenation. Nat. Rev. Mol. Cell Biol..

[87] Saul, D. & Kosinsky, R. L. Epigenetics of aging and aging-associated diseases. *Int. J. Mol. Sci*. **22**, 401 (2021). 10.3390/ijms22010401 PMC7794926 33401659

[88] Sarg B (2002). Postsynthetic trimethylation of histone H4 at lysine 20 in mammalian tissues is associated with aging. J. Biol. Chem..

[89] Liu L (2013). Chromatin modifications as determinants of muscle stem cell quiescence and chronological aging. Cell Rep..

[90] Henikoff S, Smith MM (2015). Histone variants and epigenetics. Cold Spring Harb. Perspect. Biol..

[91] Lee JH, Kim EW, Croteau DL, Bohr VA (2020). Heterochromatin: an epigenetic point of view in aging. Exp. Mol. Med..

[92] Boehm M, Slack F (2005). A developmental timing microRNA and its target regulate life span in C. elegans. Science.

[93] Lindsay M (2008). A. microRNAs and the immune response. Trends Immunol..

[94] Czesnikiewicz-Guzik M (2008). T cell subset-specific susceptibility to aging. Clin. Immunol..

[95] Manser AR, Uhrberg M (2016). Age-related changes in natural killer cell repertoires: impact on NK cell function and immune surveillance. Cancer Immunol. Immunother..

[96] Campos C (2015). Expression of NKp30, NKp46 and DNAM-1 activating receptors on resting and IL-2 activated NK cells from healthy donors according to CMV-serostatus and age. Biogerontology.

[97] Mantovani A (2004). The chemokine system in diverse forms of macrophage activation and polarization. Trends Immunol..

[98] Fontana L (2013). Aging promotes the development of diet-induced murine steatohepatitis but not steatosis. Hepatology.

[99] Lumeng CN (2011). Aging is associated with an increase in T cells and inflammatory macrophages in visceral adipose tissue. J. Immunol..

[100] Wang Y, Wehling-Henricks M, Samengo G, Tidball JG (2015). Increases of M2a macrophages and fibrosis in aging muscle are influenced by bone marrow aging and negatively regulated by muscle-derived nitric oxide. Aging Cell.

[101] Kelly J (2007). Senescence regulates macrophage activation and angiogenic fate at sites of tissue injury in mice. J. Clin. Invest..

[102] Hall BM (2017). p16(Ink4a) and senescence-associated beta-galactosidase can be induced in macrophages as part of a reversible response to physiological stimuli. Aging.

[103] Gon Y (1996). Lower serum concentrations of cytokines in elderly patients with pneumonia and the impaired production of cytokines by peripheral blood monocytes in the elderly. Clin. Exp. Immunol..

[104] Plowden J (2004). Impaired antigen-induced CD8+ T cell clonal expansion in aging is due to defects in antigen presenting cell function. Cell Immunol..

[105] van Duin D (2007). Prevaccine determination of the expression of costimulatory B7 molecules in activated monocytes predicts influenza vaccine responses in young and older adults. J. Infect. Dis..

[106] Rogers J, Rovigatti U (1988). Immunologic and tissue culture approaches to the neurobiology of aging. Neurobiol. Aging.

[107] Liang S (2009). Age-related alterations in innate immune receptor expression and ability of macrophages to respond to pathogen challenge in vitro. Mech. Ageing Dev..

[108] Jackaman C (2013). Targeting macrophages rescues age-related immune deficiencies in C57BL/6J geriatric mice. Aging Cell.

[109] Sharma S, Dominguez AL, Lustgarten J (2006). High accumulation of T regulatory cells prevents the activation of immune responses in aged animals. J. Immunol..

[110] Tu W, Rao S (2016). Mechanisms underlying T cell immunosenescence: aging and cytomegalovirus infection. Front. Microbiol..

[111] Huff, W. X. et al. The evolving role of CD8(+)CD28(-) immunosenescent T cells in cancer immunology. *Int J Mol Sci*. **20**, 2810 (2019). 10.3390/ijms20112810 PMC6600236 31181772

[112] Brenchley JM (2003). Expression of CD57 defines replicative senescence and antigen-induced apoptotic death of CD8+ T cells. Blood.

[113] Heffner M, Fearon DT (2007). Loss of T cell receptor-induced Bmi-1 in the KLRG1(+) senescent CD8(+) T lymphocyte. Proc. Natl Acad. Sci. USA.

[114] Mondal AM (2013). p53 isoforms regulate aging- and tumor-associated replicative senescence in T lymphocytes. J. Clin. Invest..

[115] Akbar AN, Henson SM (2011). Are senescence and exhaustion intertwined or unrelated processes that compromise immunity?. Nat. Rev. Immunol..

[116] Wherry EJ (2011). T cell exhaustion. Nat. Immunol..

[117] Effros RB (2011). Telomere/telomerase dynamics within the human immune system: effect of chronic infection and stress. Exp. Gerontol..

[118] Farber DL, Yudanin NA, Restifo NP (2014). Human memory T cells: generation, compartmentalization and homeostasis. Nat. Rev. Immunol..

[119] Bantug GR, Galluzzi L, Kroemer G, Hess C (2018). The spectrum of T cell metabolism in health and disease. Nat. Rev. Immunol..

[120] Nicoli F (2022). Altered basal lipid metabolism underlies the functional impairment of naive CD8(+) T cells in elderly humans. J. Immunol..

[121] Balyan, R., Gautam, N. & Gascoigne, N. R. J. The ups and downs of metabolism during the lifespan of a T cell. *Int. J. Mol. Sci*. **21**, 7972 (2020). 10.3390/ijms21217972 PMC7663011 33120978

[122] Corre I, Gomez M, Vielkind S, Cantrell DA (2001). Analysis of thymocyte development reveals that the GTPase RhoA is a positive regulator of T cell receptor responses in vivo. J. Exp. Med..

[123] Ron-Harel N (2016). Mitochondrial biogenesis and proteome remodeling promote one-carbon metabolism for T cell activation. Cell Metab..

[124] Wang R (2011). The transcription factor Myc controls metabolic reprogramming upon T lymphocyte activation. Immunity.

[125] D’Souza AD, Parikh N, Kaech SM, Shadel GS (2007). Convergence of multiple signaling pathways is required to coordinately up-regulate mtDNA and mitochondrial biogenesis during T cell activation. Mitochondrion.

[126] Ron-Harel N (2018). Defective respiration and one-carbon metabolism contribute to impaired naïve T cell activation in aged mice. Proc. Natl Acad. Sci. USA.

[127] Cui W, Kaech S (2010). Generation of effector CD8+ T cells and their conversion to memory T cells. Immunol. Rev..

[128] Chi H (2012). Regulation and function of mTOR signalling in T cell fate decisions. Nat. Rev. Immunol..

[129] Ciofani M, Zuniga-Pflucker JC (2005). Notch promotes survival of pre-T cells at the beta-selection checkpoint by regulating cellular metabolism. Nat. Immunol..

[130] Frauwirth K (2002). The CD28 signaling pathway regulates glucose metabolism. Immunity.

[131] Rao RR, Li Q, Odunsi K, Shrikant PA (2010). The mTOR kinase determines effector versus memory CD8+ T cell fate by regulating the expression of transcription factors T-bet and Eomesodermin. Immunity.

[132] Pollizzi KN (2015). mTORC1 and mTORC2 selectively regulate CD8(+) T cell differentiation. J. Clin. Invest..

[133] Zhao Y, Shao Q, Peng G (2020). Exhaustion and senescence: two crucial dysfunctional states of T cells in the tumor microenvironment. Cell Mol. Immunol..

[134] Gomes AP (2013). Declining NAD(+) induces a pseudohypoxic state disrupting nuclear-mitochondrial communication during aging. Cell.

[135] Mittelbrunn M, Kroemer G (2021). Hallmarks of T cell aging. Nat. Immunol..

[136] Yang OO (2005). Decreased perforin and granzyme B expression in senescent HIV-1-specific cytotoxic T lymphocytes. Virology.

[137] Debacq-Chainiaux F, Erusalimsky JD, Campisi J, Toussaint O (2009). Protocols to detect senescence-associated beta-galactosidase (SA-betagal) activity, a biomarker of senescent cells in culture and in vivo. Nat. Protoc..

[138] Henning AN, Klebanoff CA, Restifo NP (2018). Silencing stemness in T cell differentiation. Science.

[139] Chou C (2014). c-Myc-induced transcription factor AP4 is required for host protection mediated by CD8+ T cells. Nat. Immunol..

[140] Abdelsamed HA (2017). Human memory CD8 T cell effector potential is epigenetically preserved during in vivo homeostasis. J. Exp. Med..

[141] Akondy RS (2017). Origin and differentiation of human memory CD8 T cells after vaccination. Nature.

[142] Zediak VP, Wherry EJ, Berger SL (2011). The contribution of epigenetic memory to immunologic memory. Curr. Opin. Genet. Dev..

[143] Russ BE (2014). Distinct epigenetic signatures delineate transcriptional programs during virus-specific CD8(+) T cell differentiation. Immunity.

[144] Petkovich DA (2017). Using DNA Methylation Profiling to Evaluate Biological Age and Longevity Interventions. Cell Metab..

[145] O’Sullivan RJ, Kubicek S, Schreiber SL, Karlseder J (2010). Reduced histone biosynthesis and chromatin changes arising from a damage signal at telomeres. Nat. Struct. Mol. Biol..

[146] De Cecco M (2013). Genomes of replicatively senescent cells undergo global epigenetic changes leading to gene silencing and activation of transposable elements. Aging Cell.

[147] Funayama R, Saito M, Tanobe H, Ishikawa F (2006). Loss of linker histone H1 in cellular senescence. J. Cell Biol..

[148] Heyn H (2012). Distinct DNA methylomes of newborns and centenarians. Proc. Natl Acad. Sci. USA.

[149] Rendeiro AF (2016). Chromatin accessibility maps of chronic lymphocytic leukaemia identify subtype-specific epigenome signatures and transcription regulatory networks. Nat. Commun..

[150] Ohyashiki M (2011). Age-related decrease of miRNA-92a levels in human CD8+ T-cells correlates with a reduction of naive T lymphocytes. Immun. Ageing.

[151] Brunner S (2012). Upregulation of miR-24 is associated with a decreased DNA damage response upon etoposide treatment in highly differentiated CD8(+) T cells sensitizing them to apoptotic cell death. Aging Cell.

[152] Mogilenko DA (2021). Comprehensive profiling of an aging immune system reveals clonal GZMK(+) CD8(+) T cells as conserved hallmark of inflammaging. Immunity.

[153] Moskowitz, D. M. et al. Epigenomics of human CD8 T cell differentiation and aging. *Sci Immunol*. **2**, eaag0192 (2017). 10.1126/sciimmunol.aag0192 PMC5399889 28439570

[154] Wang SS (2019). Tumor-infiltrating B cells: their role and application in anti-tumor immunity in lung cancer. Cell Mol. Immunol..

[155] Bulati M, Caruso C, Colonna-Romano G (2017). From lymphopoiesis to plasma cells differentiation, the age-related modifications of B cell compartment are influenced by “inflamm-ageing”. Ageing Res. Rev..

[156] Frasca D (2007). Tristetraprolin, a negative regulator of mRNA stability, is increased in old B cells and is involved in the degradation of E47 mRNA. J. Immunol..

[157] Kogut I, Scholz JL, Cancro MP, Cambier JC (2012). B cell maintenance and function in aging. Semin. Immunol..

[158] Cepeda S (2018). Age-associated decline in thymic B cell expression of aire and aire-dependent self-antigens. Cell Rep..

[159] Agrawal A, Gupta S (2011). Impact of aging on dendritic cell functions in humans. Ageing Res. Rev..

[160] Salminen A, Kauppinen A, Kaarniranta K (2019). AMPK activation inhibits the functions of myeloid-derived suppressor cells (MDSC): impact on cancer and aging. J. Mol. Med..

[161] Biran A (2017). Quantitative identification of senescent cells in aging and disease. Aging Cell.

[162] Salminen A, Kauppinen A, Kaarniranta K (2018). Myeloid-derived suppressor cells (MDSC): an important partner in cellular/tissue senescence. Biogerontology.

[163] Hernandez-Segura A (2017). Unmasking transcriptional heterogeneity in senescent cells. Curr. Biol..

[164] Sheppard KA (2004). PD-1 inhibits T-cell receptor induced phosphorylation of the ZAP70/CD3zeta signalosome and downstream signaling to PKCtheta. FEBS Lett..

[165] Braumuller H (2013). T-helper-1-cell cytokines drive cancer into senescence. Nature.

[166] Collado M, Blasco MA, Serrano M (2007). Cellular senescence in cancer and aging. Cell.

[167] Wang L, Lankhorst L, Bernards R (2022). Exploiting senescence for the treatment of cancer. Nat. Rev. Cancer.

[168] Borodkina AV, Deryabin PI, Giukova AA, Nikolsky NN (2018). “Social life” of senescent cells: what is SASP and why study it?. Acta Nat..

[169] da Silva PFL (2019). The bystander effect contributes to the accumulation of senescent cells in vivo. Aging Cell.

[170] Childs BG (2016). Senescent intimal foam cells are deleterious at all stages of atherosclerosis. Science.

[171] Grahame-Clarke C (2003). Human cytomegalovirus seropositivity is associated with impaired vascular function. Circulation.

[172] Yu TH (2015). Characterization of CD8(+)CD57(+) T cells in patients with acute myocardial infarction. Cell Mol. Immunol..

[173] Luz Correa B (2014). The inverted CD4:CD8 ratio is associated with cytomegalovirus, poor cognitive and functional states in older adults. Neuroimmunomodulation.

[174] Bauer ME (2020). Accelerated immunosenescence in rheumatoid arthritis: impact on clinical progression. Immun. Ageing.

[175] Martens PB, Goronzy JJ, Schaid D, Weyand CM (1997). Expansion of unusual CD4+ T cells in severe rheumatoid arthritis. Arthritis Rheum..

[176] Sallusto F (1999). Two subsets of memory T lymphocytes with distinct homing potentials and effector functions. Nature.

[177] Goronzy JJ (2004). Prognostic markers of radiographic progression in early rheumatoid arthritis. Arthritis Rheum..

[178] Scarsi M, Ziglioli T, Airo P (2010). Decreased circulating CD28-negative T cells in patients with rheumatoid arthritis treated with abatacept are correlated with clinical response. J. Rheumatol..

[179] Prelog M (2006). Aging of the immune system: a risk factor for autoimmunity?. Autoimmun. Rev..

[180] Lindqvist D (2013). Cerebrospinal fluid inflammatory markers in Parkinson’s disease-associations with depression, fatigue, and cognitive impairment. Brain Behav. Immun..

[181] Ebbert MTW (2017). Conserved DNA methylation combined with differential frontal cortex and cerebellar expression distinguishes C9orf72-associated and sporadic ALS, and implicates SERPINA1 in disease. Acta Neuropathol..

[182] Bhat R (2012). Astrocyte senescence as a component of Alzheimer’s disease. PLoS ONE.

[183] Saez-Atienzar S, Masliah E (2020). Cellular senescence and Alzheimer disease: the egg and the chicken scenario. Nat. Rev. Neurosci..

[184] Lu T (2016). Addendum: REST and stress resistance in ageing and Alzheimer’s disease. Nature.

[185] Lu T (2014). REST and stress resistance in ageing and Alzheimer’s disease. Nature.

[186] Pelissier Vatter FA (2018). High-dimensional phenotyping identifies age-emergent cells in human mammary epithelia. Cell Rep..

[187] Gomes AP (2020). Age-induced accumulation of methylmalonic acid promotes tumour progression. Nature.

[188] Ecker BL (2019). Age-related changes in HAPLN1 increase lymphatic permeability and affect routes of melanoma metastasis. Cancer Discov..

[189] Ershler WB, Socinski MA, Greene CJ (1983). Bronchogenic cancer, metastases, and aging. J. Am. Geriatr. Soc..

[190] Fisher CJ (1997). Histopathology of breast cancer in relation to age. Br. J. Cancer.

[191] Chen Y (2021). Aging in COVID-19: vulnerability, immunity and intervention. Ageing Res. Rev..

[192] Li J (2020). Clinical features of familial clustering in patients infected with 2019 novel coronavirus in Wuhan, China. Virus Res..

[193] Salminen A (2021). Feed-forward regulation between cellular senescence and immunosuppression promotes the aging process and age-related diseases. Ageing Res. Rev..

[194] van Deursen JM (2014). The role of senescent cells in ageing. Nature.

[195] Lee S, Schmitt C (2019). The dynamic nature of senescence in cancer. Nat. Cell Biol..

[196] Patel PL (2016). Derepression of hTERT gene expression promotes escape from oncogene-induced cellular senescence. Proc. Natl Acad. Sci. USA.

[197] Mannick, J. B. et al. TORC1 inhibition enhances immune function and reduces infections in the elderly. *Sci. Transl. Med*. **10**, eaaq1564 (2018). 10.1126/scitranslmed.aaq1564 29997249

[198] Giallongo C (2018). Monocytic myeloid-derived suppressor cells as prognostic factor in chronic myeloid leukaemia patients treated with dasatinib. J. Cell. Mol. Med..

[199] Kishton RJ, Sukumar M, Restifo NP (2017). Metabolic regulation of T cell longevity and function in tumor immunotherapy. Cell Metab..

[200] Morgan RA (2006). Cancer regression in patients after transfer of genetically engineered lymphocytes. Science.

[201] Vigano S, Perreau M, Pantaleo G, Harari A (2012). Positive and negative regulation of cellular immune responses in physiologic conditions and diseases. Clin. Dev. Immunol..

[202] Ferrara R (2017). Immunosenescence and immunecheckpoint inhibitors in non-small cell lung cancer patients: Does age really matter?. Cancer Treat. Rev..

[203] Amor C (2020). Senolytic CAR T cells reverse senescence-associated pathologies. Nature.

[204] Baker DJ (2011). Clearance of p16Ink4a-positive senescent cells delays ageing-associated disorders. Nature.

[205] Baker DJ (2016). Naturally occurring p16(Ink4a)-positive cells shorten healthy lifespan. Nature.

[206] Li L (2019). TLR8-mediated metabolic control of human treg function: a mechanistic target for cancer immunotherapy. Cell Metab..

[207] Zhan JK (2018). AMPK/TSC2/mTOR pathway regulates replicative senescence of human vascular smooth muscle cells. Exp. Ther. Med..

[208] Phan AT, Goldrath AW, Glass CK (2017). Metabolic and epigenetic coordination of T cell and macrophage immunity. Immunity.

[209] Li G (2012). Decline in miR-181a expression with age impairs T cell receptor sensitivity by increasing DUSP6 activity. Nat. Med..

[210] Li QJ (2007). miR-181a is an intrinsic modulator of T cell sensitivity and selection. Cell.

[211] Hu B (2020). Distinct age-related epigenetic signatures in CD4 and CD8 T cells. Front Immunol..

[212] Arts RJ (2016). Glutaminolysis and fumarate accumulation integrate immunometabolic and epigenetic programs in trained immunity. Cell Metab..

[213] McGuire, P. J. Mitochondrial dysfunction and the aging immune system. *Biology* **8**, 26 (2019). 10.3390/biology8020026 PMC6627503 31083529

[214] McCay CM, Maynard LA, Sperling G, Barnes LL (1975). The Journal of Nutrition. Volume 18 July–December, 1939. Pages 1–13. Retarded growth, life span, ultimate body size and age changes in the albino rat after feeding diets restricted in calories. Nutr. Rev..

[215] Saçma M, Geiger H (2021). Exercise generates immune cells in bone. Nature.

[216] Di Biase S (2016). Fasting-mimicking diet reduces HO-1 to promote T cell-mediated tumor cytotoxicity. Cancer Cell.

[217] Druzd D (2017). Lymphocyte circadian clocks control lymph node trafficking and adaptive immune responses. Immunity.

[218] Pietrocola F (2016). Caloric restriction mimetics enhance anticancer immunosurveillance. Cancer Cell.

[219] Honda K, Littman DR (2016). The microbiota in adaptive immune homeostasis and disease. Nature.

[220] Butler T, Gibbs J (2020). Circadian host-microbiome interactions in immunity. Front. Immunol..

[221] Moller SH (2022). Metabolic programs tailor T cell immunity in viral infection, cancer, and aging. Cell Metab..

[222] Berger, S. & Sassone-Corsi, P. Metabolic signaling to chromatin. *Cold Spring Harb. Perspect. Biol.* **8**, a019463 (2016). 10.1101/cshperspect.a019463 PMC5088527 26492570

[223] Querec TD (2009). Systems biology approach predicts immunogenicity of the yellow fever vaccine in humans. Nat. Immunol..

[224] Nakaya HI (2011). Systems biology of vaccination for seasonal influenza in humans. Nat. Immunol..

[225] Zimmermann MT (2016). System-wide associations between DNA-methylation, gene expression, and humoral immune response to influenza vaccination. PLoS ONE.

[226] Chou J, Effros R (2013). T cell replicative senescence in human aging. Curr. Pharm. Des..

[227] Haralambieva IH (2015). The impact of immunosenescence on humoral immune response variation after influenza A/H1N1 vaccination in older subjects. PLoS ONE.

[228] Tan Y (2014). Gene signatures related to B-cell proliferation predict influenza vaccine-induced antibody response. Eur. J. Immunol..

[229] Oh JZ (2014). TLR5-mediated sensing of gut microbiota is necessary for antibody responses to seasonal influenza vaccination. Immunity.

[230] Liu Y (2009). Expression of p16(INK4a) in peripheral blood T-cells is a biomarker of human aging. Aging Cell.

[231] Bernardes de Jesus B, Blasco MA (2012). Assessing cell and organ senescence biomarkers. Circ. Res..

[232] Reiser J, Banerjee A (2016). Effector, memory, and dysfunctional CD8(+) T cell fates in the antitumor immune response. J. Immunol. Res..

[233] Wherry EJ (2007). Molecular signature of CD8+ T cell exhaustion during chronic viral infection. Immunity.

[234] Blackburn SD (2009). Coregulation of CD8+ T cell exhaustion by multiple inhibitory receptors during chronic viral infection. Nat. Immunol..

[235] Canale FP (2018). CD39 expression defines cell exhaustion in tumor-infiltrating CD8(+) T cells-response. Cancer Res..

[236] Williams J (2017). The EGR2 targets LAG-3 and 4-1BB describe and regulate dysfunctional antigen-specific CD8+ T cells in the tumor microenvironment. J. Exp. Med..

[237] Kasakovski D, Xu L, Li Y (2018). T cell senescence and CAR-T cell exhaustion in hematological malignancies. J. Hematol. Oncol..

[238] Nguyen V, Mendelsohn A, Larrick JW (2017). Interleukin-7 and Immunosenescence. J. Immunol. Res..

[239] Sen DR (2016). The epigenetic landscape of T cell exhaustion. Science.

[240] Ghoneim HE (2017). De novo epigenetic programs inhibit PD-1 blockade-mediated T cell rejuvenation. Cell.

